# Large-scale insect outbreak homogenizes the spatial structure of ectomycorrhizal fungal communities

**DOI:** 10.7717/peerj.6895

**Published:** 2019-05-10

**Authors:** Gregory J. Pec, James F. Cahill, Jr.

**Affiliations:** 1 Department of Natural Resources and the Environment, University of New Hampshire, Durham, NH, United States of America; 2 Department of Biological Sciences, University of Alberta, Edmonton, Canada

**Keywords:** Biotic disturbance, Community structure, Dendroctonus ponderosae, Pinus contorta, Ion torrent, Ectomycorrhizal fungi, Semivariograms

## Abstract

Ectomycorrhizal fungi (plant symbionts) are diverse and exist within spatially variable communities that play fundamental roles in the functioning of terrestrial ecosystems. However, the underlying ecological mechanisms that maintain and regulate the spatial structuring of ectomycorrhizal fungal communities are both complex and remain poorly understood. Here, we use a gradient of mountain pine beetle (*Dendroctonus ponderosae*) induced tree mortality across eleven stands in lodgepole pine (*Pinus contorta*) forests of western Canada to investigate: (i) the degree to which spatial structure varies within this fungal group, and (ii) how these patterns may be driven by the relative importance of tree mortality from changes in understory plant diversity, productivity and fine root biomass following tree death. We found that the homogeneity of the ectomycorrhizal fungal community increased with increasing tree death, aboveground understory productivity and diversity. Whereas, the independent effect of fine root biomass, which declined along the same gradient of tree mortality, increased the heterogeneity of the ectomycorrhizal fungal community. Together, our results demonstrate that large-scale biotic disturbance homogenizes the spatial patterns of ectomycorrhizal fungal communities.

## Introduction

The spatial structure of ecological communities can arise from both deterministic and neutral processes (Peres-Neto & Legendre, 2010). For example, differences in the distribution of ecological communities can derive from differences in the habitat requirements of individual species resulting in a filtering or sorting process (Cottenie, 2005). Moreover, differences in the spatial patterning of ecological communities may also reflect the importance of neutral processes such as dispersal limitation (Lekberg et al., 2007), priority effects or chance events (Fukami, 2015). The roles of both these processes individually or in combination can influence the spatial patterning of ecological communities (Chase & Myers, 2011; Soininen, 2016), which can ultimately lead to changes in the functioning of terrestrial ecosystems, such as in aboveground plant productivity and regeneration (Van Der Heijden, Bardgett & Van Straalen, 2008; Spasojevic & Suding, 2012; Kardol, Souza & Classen, 2013) nutrient cycling and decomposition rates of organic matter in soils (Baldrian et al., 2013; Bardgett & Van der Putten, 2014; Kyaschenko et al., 2017). Despite an increased awareness of the relative influence of biological processes in shaping the spatial structure of animal and plant communities (Nekola & White, 1999; Condit et al., 2002; Poulin, 2003), the underlying ecological mechanisms responsible for determining the spatial structuring of microbial communities, particularly ectomycorrhizal (EM) fungi, requires further study (Ettema & Wardle, 2002; Green et al., 2004; Green & Bohannan, 2006; Robinson et al., 2009; Pickles & Anderson, 2016).

Ectomycorrhizal fungi, primary plant symbionts in terrestrial ecosystems (Smith & Read, 2008), play vital roles in ecosystem functions such as carbon flow and nutrient cycling as well as in forest regeneration and succession (Read & Perez-Moreno, 2003; Smith & Read, 2008; Clemmensen et al., 2013). Individual EM fungi, EM fungal species, and communities of EM fungi can exhibit variability in spatial structure over scales ranging from a few centimeters to hundreds of kilometers (Hallenberg & Kúffer, 2001; Lilleskov et al., 2004; Peay et al., 2007; Pickles et al., 2010; Pickles et al., 2012; Bahram et al., 2013). Along with spatial variability in the community composition of EM fungi, several biotic factors such as host species presence and identity, and plant diversity and productivity, also show considerable spatial variability ranging at the scale of centimeters to hundreds of meters (Saetre & Bååth, 2000; Ettema & Wardle, 2002; Tedersoo et al., 2014; Pec et al., 2015; Sorenson et al., 2017). However, the extent to which the spatial structuring of EM fungi is influenced by variation within the biotic environment remains unclear. Here, our objectives were to determine: (i) the degree to which spatial structure varies within this fungal group, and (ii) how these patterns may be driven by the relative importance of tree mortality from changes in understory plant diversity, productivity and fine root biomass following tree death. Changes to the spatial structuring of these taxa, for example, can have potential consequences for forest regeneration, forest succession, and cause shifts in nutrient availability for aboveground vegetation (Jones, Durall & Cairney, 2003; Izzo, Agbowo & Bruns, 2005; Baldrian et al., 2013; Kyaschenko et al., 2017).

Our earlier study (Pec et al., 2017) evaluated the effects of tree mortality, soils, and geographical distance on the richness and composition of soil fungi following a mountain pine beetle outbreak. Here, we include and re-analyze a subset of that dataset, along with additional spatially explicit sampling, and geostatistics to explore if biotic factors (variability in plant diversity, plant productivity and fine root biomass following host-tree mortality) disrupt the spatial structure of EM fungi. Although the spatial distribution of EM fungi is affected by multiple factors such as variability in soil nutrients, moisture, organic matter and temperature (Tedersoo et al., 2012; Tedersoo et al., 2014; Bahram et al., 2013), the identities of the species making up the plant community are also important in determining the identities of the species in the ectomycorrhizal fungi community (Ishida, Nara & Hogetsu, 2007). Our previous research has shown that the death of trees coincides with variation in plant diversity and productivity at multiple spatial scales (Pec et al., 2015; Cigan et al., 2015). Specifically, stands with widespread mortality of lodgepole pine (*Pinus contorta* Dougl. ex. Loud. var. *latifolia* Engelm.), as compared to undisturbed stands, showed a decline in overall fine root biomass and an increase in the productivity and diversity of understory vegetation. Thus, variation in biotic conditions may lead to differences, or a lack thereof, in the scale of patchiness for communities of EM fungi.

## Materials & Methods

### Study area

Eleven forest stands were located within a 625-km^2^ region experiencing mountain pine beetle activity since 2009 within the Lower Foothills southwest of Grande Prairie, Alberta (54°39′N, 118°59′W; 950–1,150 m above sea level). Canopies were dominated (≥80%) by even-aged (120 ± 0.4 SE years old) lodgepole pine, and across stands a gradient of beetle-induced tree mortality was captured (0 to 82% lodgepole pine basal area killed) (Cigan et al., 2015). Within stands, *Abies balsamea* (L.) Mill, *Betula papyrifera* Marshall, *Picea glauca* (Moench) Voss, *Picea mariana* Mill. Britton, Sterns, & Pogenb., and *Populus tremuloides* Michx. were interspersed in the subcanopy (0 to 14% of total basal area) along with a mixture of mostly herbaceous (e.g., *Chamerion angustifolium* (L.)) and to a lesser extent woody (e.g., *Vaccinium* spp.) vegetation in the understory (Pec et al., 2015). Soils were classified as Orthic Gray Luvisols derived from imperfectly drained glacial tills (Soil Classification Working Group, 1998). Detailed information on stand selection and description, including stand locations and structure, is presented in Treu et al. (2014) and Cigan et al. (2015).

### Fungal sampling

In May–June 2012, we established a 1,600-m^2^ (40 m × 40 m) plot within each of the eleven stands and ten 9 m × 9 m subplots within each plot. Within each of the 110 subplots, eight soil cores (5 cm diameter, 20 cm deep) were positioned at distances (0.5 m, 1 m, 1.5 m, 2 m, 3 m, 4 m, 5 m) randomly radiating from the center of each subplot (Fig. S1). In total, 880 soil cores were sampled for fungi found on fine roots and in soils. Geographical coordinates (Garmin GPSmap 60Cx; Garmin International, Olathe, KS, USA) were also recorded at each sampled soil core.

### Biotic drivers

To determine the effect of tree mortality following mountain pine beetle outbreak on the spatial structure of EM fungi, we also recorded diameter at breast height (≥1.3 m), species identity, and health status of all mature pine trees, and breast height and species identity of all subordinate tree species within each subplot in June 2012. Attack by mountain pine beetle on mature lodgepole pine trees was confirmed by the presence of pitch tubes, boring dust, exit holes, and subcortical galleries. Tree mortality was calculated as lodgepole pine basal area killed divided by the total basal area of all trees, expressed as a percentage for each subplot. Subplot values were averaged to generate estimates of tree mortality for each plot.

To determine understory plant diversity, we identified all herbaceous and woody species within a 1 m × 1 m quadrat near the center of each subplot in June 2012 (see Pec et al. (2015) for a detailed list). To determine understory biomass, we harvested all aboveground parts of the understory vegetation by species from each 1 m × 1 m quadrat in August 2012. Harvested plants were dried at 70 °C for 48 h, weighed, and averaged for each plot. To determine the effect of belowground fine root biomass on the spatial structure of EM fungi, we extracted soil cores (5 cm diameter, 20 cm deep) next to each 1 m × 1m quadrat. Roots were washed over a 2 mm sieve and living roots were distinguished from dead roots based on the integrity and color of vascular tissue. Fine roots (<2 mm) as well as any higher order roots were dried at 60 °C for 48 h, weighed, and values were averaged for each plot.

### Molecular characterization of fungi

Fungi occurring on roots and in soils were sampled from the soil cores described above. In total, 880 samples (8 soil cores ×10 subplots × 11 plots) were transported on ice and frozen at −20 °C until processed. Soil samples were thawed and subsamples of 250 mg of soil with roots of all species included were placed in a pre-chilled freeze-dryer (VirTis Freezermobile FM25XL; SP Scientific, Warminster, PA, USA) at −45 °C and lyophilized for 24 h. Soils were homogenized using a mixer mill (Retsch Type MM 301; Retsch GmbH, Haan, Germany). Genomic DNA was isolated from 250 mg of ground soil using the CTAB technique of Pec et al. (2017). In brief, CTAB buffer (700 µl) and 10 µl of proteinase K (600 mAU ml^−1^; Qiagen Inc., Mississauga, Ontario, Canada) were added to each sample. Samples were incubated at 65 °C for 1 h, cooled to 21 °C, and 600 µl of 24:1 chloroform-isoamyl alcohol were added. Samples were centrifuged for 5 min at 17,000 g and 21 °C followed by 600 µl isopropanol at −20 °C for 2 h. Samples were centrifuged for 15 min, supernatant was discarded followed 500 µl of 95% ethanol (v/v) added to pellet, vortexed and centrifuged for 3 min. This was repeated with 500 µl of 70% ethanol (v/v) followed by pellets being resuspended in 50 µl nuclease-free water (Life Technologies, Carlsbad, CA, USA).

PCR amplification was performed to amplify the internal transcribed spacer (ITS) 1 region of nuclear rDNA using primers ITS1F and ITS2 with mixtures containing the following: 19.0 µl of Platinum PCR SuperMix High Fidelity (Invitrogen; Life Technologies, Carlsbad, California, USA), 0.5 µl of 10 µM forward primer, 0.5 µl of 10 µM reverse primer, and 5 µl of DNA template (see Table S1 for a list of Ion Torrent™ adaptor, primer, and specific multiplex identifier barcode sequences). Negative (5 µl of sterile water) and positive control (5 µl of *Agaricus bisporus* DNA) reactions contained the same mixtures replacing the DNA template. Thermocycler conditions used for PCR amplification were as follows: one cycle of 94 °C for 2 min, then 30 cycles of 94 °C for 30 s, 45 °C for 30 s, 68 °C for 60 s, and ending with one cycle for 68 °C for 7 min. Gel electrophoresis was used to confirm successful amplification. Bands between 150–400 bp were excised from the gel, PCR products were purified using Qiaquick gel extraction kit (Qiagen Inc., Mississauga, Ontario, Canada), quantified fluorescently using a dsDNA HS assay kit on a Qubit fluorometer (Invitrogen, Carlsbad, CA, USA), and pooled into equimolar concentrations. A second gel extraction cleanup was conducted on the pooled products, quantified, and diluted prior to emulsion PCR. An emulsion PCR quality check was conducted prior to sequencing using an Ion OneTouch™ system (Life Technologies, Carlsbad, CA, USA). Amplicon library sequencing was performed on an Ion Torrent™ PGM 400 Sequencing Kit and Ion 316™ Chips (Life Technologies, Carlsbad, CA, USA) at the Molecular Biological Sciences Facility, University of Alberta.

### Bioinformatic analysis

Initial sequence processing of Ion Torrent™ data (∼400 bp) was performed using Trimmomatic v0.36 (Bolger, Lohse & Usadel, 2014), removing Ion Torrent™ adapters, sequences <200 bp, and quality scores <25. Of the sequence pool generated, we detected 0% of samples from negative controls following initial quality filtering. Error rate, the variable determining the specificity in which reads can be classified, for Ion Torrent ^TM^ sequencing averaged 1.4 errors per 100 bases for read sequences which is consistent with previous observations using this sequencing platform (Salipante et al., 2014). ITS1 region was extracted using ITSx v1.0.11 (Bengtsson-Palme et al., 2013) and sequences were clustered into operational taxonomic units (OTUs) at 97% sequence similarity with removal of chimeric sequences using the UPARSE-OUT algorithm (Edgar, 2013) with the *cluster_otus* command in USEARCH (v9.2.64) (Edgar, 2010). We excluded global singletons and clusters with fewer than five reads to reduce artificially inflating richness due to sequencing error. Representative sequences were identified with the *usearch_global* command in USEARCH (v9.2.64). OTUs were taxonomically identified using the UNITE fungal ITS database with the BLAST option in the *assign_taxonomy.py* script in Qiime v1.8. OTUs were assigned to EM fungi based on their genus affiliation, trophic mode and functional guild as described in (Branco, Bruns & Singleton, 2013; Tedersoo et al., 2014) and using the FUNGuild database (Nguyen et al., 2016). As FunGuild primarily considers genus-level assignments, OTUs were placed into the EM group only if assignments were deemed as highly probable (=absolute certain) or probable (=fairly certain) based on default parameters in FunGuild. As we were interested only in EM fungi, non-fungal OTUs (0.001%) and non-EM fungal OTUs (85%) were excluded from further analyses. Assignments were checked manually for accuracy. Representative sequences of EM fungal OTUs are deposited in GenBank under accession numbers (KR584666–KR584685; KX497205–KX498025).

### Statistical analysis

Prior to statistical analyses, which were carried out using R v.3.5.1 (R Development Core Team, 2018), we applied the Shannon diversity index (*H′*) as a measure of species abundance and richness to quantify diversity of understory plant species. Sequence data were first rarefied (12,741) to account for uneven sequence depths using 1,000 iterations with the *rarefy* function in the package *vegan* (Oksanen et al., 2013). To test for changes in EM fungal OTU richness as a result of tree mortality, a generalized linear model with a Poisson distribution was performed, while a linear model was performed to test for changes in the relative sequence abundance of EM fungi following tree mortality. Data was analyzed at the plot level.

Indicator species analysis was performed to identify EM fungal OTUs, based on relative sequence abundances, that were significantly associated with undisturbed and severely beetle-killed stands using the *multipatt()* function in the R package *indispecies* (Cáceres & Legendre, 2009). Indicator species analysis incorporates two components, (i) specificity –where 1 equals a species is found exclusively in one group, and (ii) fidelity –where 1 equals a species is found in all plots of one group and no plots in any other group. To identity EM fungal OTUs common or representative of either undisturbed or severely beetle-killed forest stands, we used the multinomial species classification method in the *vegan* package (Oksanen et al., 2013) with “supermajority” rule and all other parameters set as default (i.e., CLAM test Chazdon et al., 2011) to statistically classify EM fungal OTUs into the following categories: EM fungi primarily found in undisturbed forest sites, EM fungi primarily found in beetle-killed sites, and fungi common in both undisturbed and beetle-killed forest sites.

Semivariograms were used to determine how relative sequence abundance for the entire community of EM fungi was related to distance between soil samples per plot (*n* = 11; pairwise distances per plot = 2,627) using the *variog* function in the package *geoR* (Ribeiro Jr & Diggle, 2001). Each experimental variogram provides information on the overall spatial pattern and on the estimation of spatial autocorrelation parameters: (1) variance attributed to spatial autocorrelation (C_1_); (2) variance not attributed to spatial autocorrelation, autocorrelation at finer scales than were measured, or sampling error (C_0_); (3) the proportion of variance resulting from spatial structure (C_1_/(C_0_+C_1_) with 0 indicating no measurable spatial structure and 1 indicating that all variance is caused by spatial structure; and (4) the ‘range’, or the distance at which data is no longer spatially autocorrelated.

To determine the effect of biotic factors (plant diversity, plant productivity, fine root biomass, host-tree mortality) on the spatial structuring of EM fungi multiple linear regressions were performed using the *stats* package. The variance inflation factor (VIF) was used to detect multicollinearity (>10 indicates a strong multicollinearity) (O’brien, 2007). All predictor variables were included as there were no indicators of strong multicollinearity (Range = 1.7–2.3). Data was analyzed at the plot level. We determined the most suitable model based on *r*^2^-values. The *r*^2^-value was calculated by fitting the experimental semivariograms to theoretical semivariograms (covariance functions: e.g., exponential model, spherical model) with the most suitable model fit having the greatest *r*^2^-value, an indicator of how well the experimental semivariogram fits (a) the experimental data, and (b) the theoretical semivariogram (Legendre & Legendre, 2012). All model assumptions were checked with diagnostic plots of the residuals (Zuur et al., 2009).

## Results

In total, 31,542,423 sequences were obtained across all samples. After quality filtering, 15,439,767 sequences (49%) representing 865 fungal OTUs were assessed for taxonomic affiliation. Of those, 4,751,190 sequences (31%) representing a total of 121 EM fungal OTUs were identified, with 115 OTUs belonging to the Basidiomycota (4,704,955 sequences (99% of the relative sequence adundance)) and six OTUs belonging to the Ascomycota (46,235 sequences (1% of relative sequence abundance)) (Table S2). EM fungal OTU richness declined with tree mortality (*χ*^2^ = 11.20, *P* = 0.0008; undisturbed: mean ± SE, 43 ± 4, >80% attacked: mean ± SE, 20 ± 3). Similarly, the relative sequence abundance of EM fungi declined with tree mortality (*F* = 14.00, *P* = 0.0002; undisturbed: mean ± SE, 30.9% ± 1.1, >80% attacked: mean ± SE, 23.6% ± 2.1).

There were a total of six indicator EM fungal OTUs identified across the tree mortality gradient. In particular, *Cortinarius* and *Russula* species were associated with undisturbed forests, while *Piloderma* was mainly associated with high beetle-killed sites (Table 1). Overall, 73% of EM fungal OTUs were present in both undisturbed and beetle-killed forest sites; whereas, an unequal proportion of EM fungal OTUs were found in undisturbed forests (17%, 20 EM OTUs) versus beetle-killed sites (13%, 11 EM OTUs) (Table S2).

**Table 1:** List of indicator ectomycorrhizal fungal taxa present in soil cores from undisturbed and severely beetled-killed (>60% Pinus contorta killed basal area) stands of west-central Alberta, Canada.

Tree mortality	Taxon	Indicator value
Undisturbed	*Cortinarius* sp. 3	0.67
	*Russula* sp. 20	0.67
	*Cortinarius* sp. 22	0.61
	*Russula* sp. 9	0.61
	*Russula* sp. 8	0.60
Severely attacked	*Piloderma* sp. 11	0.45

**Notes.**

All indicator taxon at *P* < 0.01. An indicator value of 1 indicates a species found in all plots of one group and no plots in any other group. *P*-values were calculated based on a Monte Carlo significance test of observed maximum indicator values for each species.

**Figure 1:**
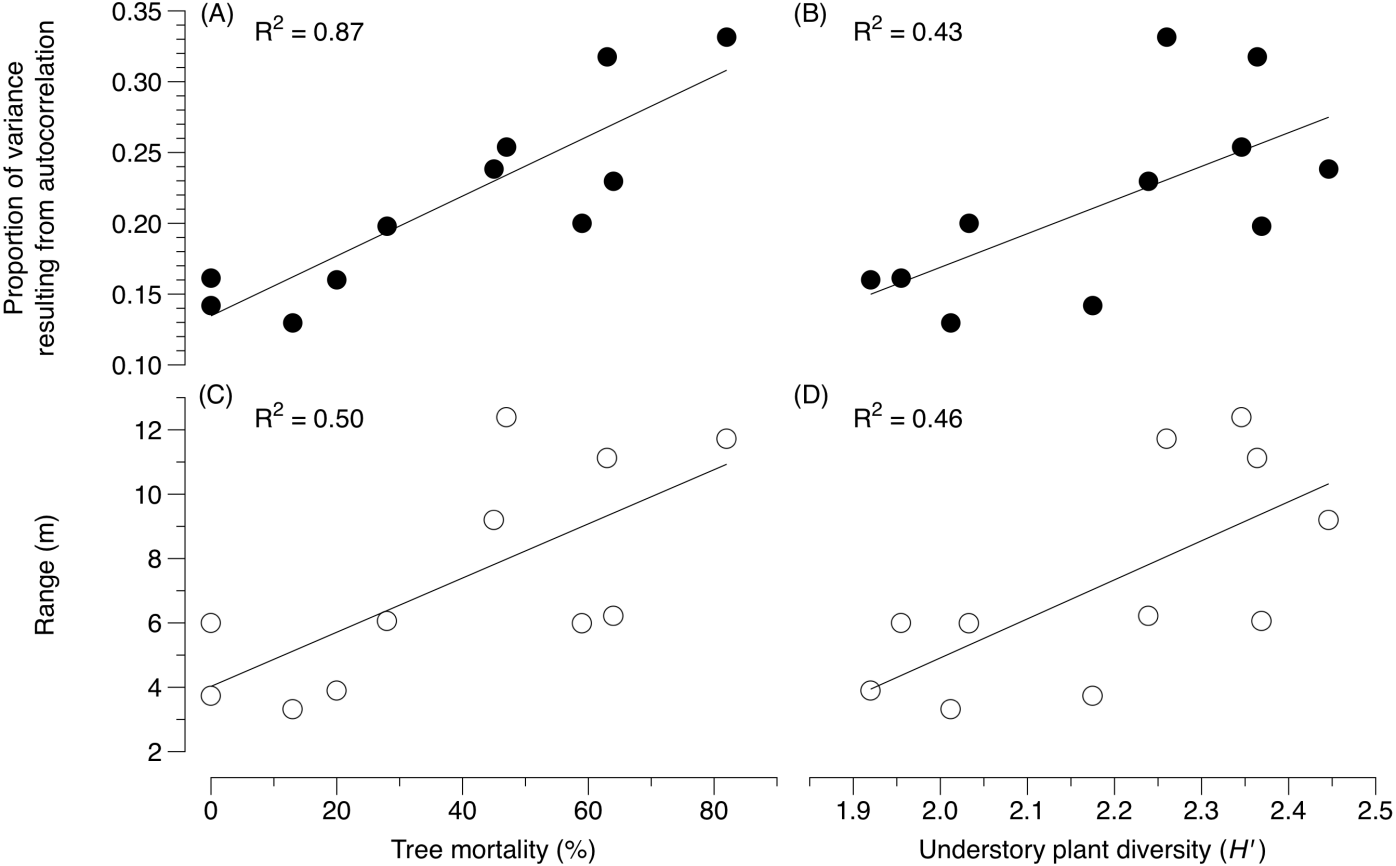
Variation in ectomycorrhizal fungal community structure as a function of tree mortality and understory plant diversity. (A) The proportion of variance due to the spatial structure of ectomycorrhizal fungal communities as a function of mountain pine beetle-induced tree mortality and (B) understory plant diversity, (C) the variation in the distance at which ectomycorrhizal fungal communities are no longer spatially autocorrelated as a function of mountain pine beetle-induced tree mortality and (D) understory plant diversity.

**Figure 2:**
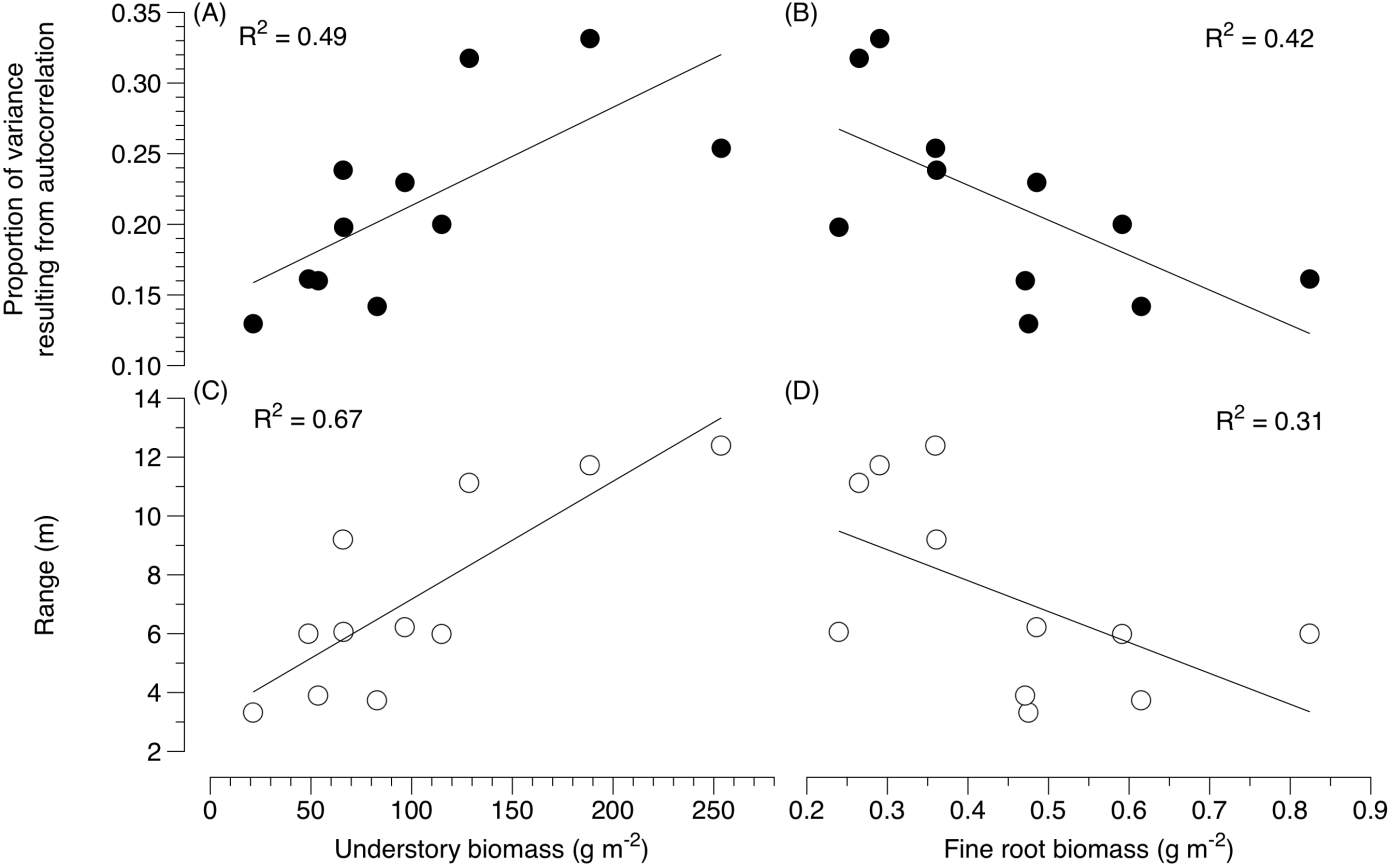
Variation in ectomycorrhizal fungal community structure as a function of aboveground understory biomass and fine root biomass. (A) The proportion of variance due to the spatial structure of ectomycorrhizal fungal communities as a function of aboveground understory biomass and (B) fine root biomass, (C) the variation in the distance at which ectomycorrhizal fungal communities are no longer spatially autocorrelated as a function of aboveground understory biomass and (D) fine root biomass.

Variation in community structure due to spatial structure varied from 13 to 33% for EM fungi across plots (Table 2). The distance over which spatial autocorrelation was detected for EM fungi ranged from 0.9 to 11.7 m across sites (Table 2). The spatial autocorrelation in EM fungal community structure (based on sequence abundance) increased with beetle-induced tree mortality (*F* = 34.20, *P* < 0.0002) (Fig. 1A). Independent of tree mortality, the proportion of variance due to autocorrelation for EM fungi also increased with plant diversity (*F* = 2.38, *P* = 0.03) (Fig. 1B) and aboveground productivity in the understory (*F* = 8.10, *P* = 0.02) (Fig. 2A), and with an overall decline in fine root biomass (*F* = 7.09 *P* = 0.03) (Fig. 2B).

**Table 2:** Semivariance analysis of the sequence abundance of ectomycorrhizal fungi along a gradient of lodgepole pine killed by mountain pine beetle (*n* = 11 sites) in pine forests of west-central Alberta, Canada.

Site ID	Tree mortality (%)	Structured variance (C)_1_^a^	Nugget variance (C_0_)^b^	Spatial structure C_1_/(C_0_+C_1_)^c^	Model fit (*r*^2^)^d^	Range (m)^e^	Covariance function^f^
1	0	0.090	0.468	0.161	0.197	6.007	Exponential model
2	0	0.084	0.508	0.142	0.238	3.743	Exponential model
3	13	0.071	0.478	0.130	0.331	3.327	Exponential model
4	28	0.174	0.705	0.198	0.231	6.068	Exponential model
5	47	0.075	0.220	0.254	0.377	12.400	Exponential model
6	20	0.085	0.446	0.160	0.140	3.906	Spherical model
7	45	0.060	0.190	0.240	0.390	9.207	Exponential model
8	59	0.010	0.040	0.200	0.149	5.998	Exponential model
9	63	0.181	0.389	0.318	0.235	11.132	Cubic model
10	64	0.161	0.540	0.230	0.422	6.228	Exponential model
11	82	0.285	0.581	0.329	0.388	11.731	Exponential model

**Notes.**

a Variance attributed to spatial autocorrelation.

b Variance not attributable to spatial autocorrelation.

c Proportion of variance due to spatial structure.

d Proportion of the total variation accounted for by fitting the experimental semivariograms to theoretical semivariograms.

e Distance at which data is no longer spatially autocorrelated.

f Represents most suitable theoretical semivariogram model to the experimental data.

The distance over which the community composition of ectomycorrhizal fungi became dissimilar (i.e., range) increased with increases in tree mortality (*F* = 8.43, *P* = 0.02) (Fig. 1C). Independent of tree mortality, the range at which ectomycorrhizal fungi became dissimilar also increased with plant diversity (*F* = 7.27, *P* = 0.02) (Fig. 1D) and aboveground productivity in the understory (*F* = 20.34, *P* = 0.002) (Fig. 2C), and a decline in fine root biomass (*F* = 5.01, *P* = 0.05) (Fig. 2D).

## Discussion

Our findings show that following large-scale biotic disturbance, the distance at which EM fungal communities became dissimilar increased with increased tree mortality, understory diversity and productivity, and loss of fine root biomass. A combination of biotic effects, representing changes in the plant community resulting from beetle-kill, had a significant impact on changes in the spatial structure of EM fungal communities. We observed that EM fungal communities became more homogeneous over larger distances in beetle-killed compared to undisturbed sites. In particular, undisturbed sites harbored EM fungi such as *Cortinarius* and *Russula* species, which is in concurrence with other studies showing an association of discrete patchiness with high mycorrhizal abundance of these types of EM fungal taxa (Lilleskov et al., 2002; Genney, Anderson & Alexander, 2006; Pickles et al., 2010). In contrast, EM species such as *Piloderma* are shown to be abundant in undisturbed coniferous forests (Dunham, Larsson & Spatafora, 2007). However, in our study, *Piloderma* strongly associated with severely beetle-killed sites and it is possible that *Piloderma* is able to take advantage of (i) resources such as N (which also increased across the tree-mortality gradient, see Cigan et al., 2015) or (ii) the competitive exclusion of weaker competitors (Sun et al., 2015).

A direct consequence of tree mortality is a severe loss in carbon flow to EM fungi (Högberg et al., 2001; Högberg & Högberg, 2002) and an increase in deadwood and substrate availability (Stursova et al., 2014), which can cause compositional shifts in EM fungal communities (Saravesi et al., 2008; Saravesi et al., 2015; Stursova et al., 2014). In addition, tree mortality in boreal forests coincides with changes in forest structure, specifically with an increase in understory diversity and productivity, due to a release from overstory competition (e.g., loss of fine root biomass) following tree death (Pec et al., 2015; Cigan et al., 2015). While the increase in understory diversity and productivity, particularly of woody perennials, may provide fine root substrate, the loss of dominant tree species (i.e., pine) can potentially increase the amount of litter and deadwood available for saprotrophic fungi (De Bellis, Kernaghan & Widden, 2007; Broeckling et al., 2008; Royer-Tardif, Bradley & Parsons, 2010). In addition, increases in understory diversity and productivity may also elevate the input of root exudates into soils, which have been shown to cause compositional shifts in soil fungal communities (Broeckling et al., 2008; Berg & Smalla, 2009).

In our study, there was also an increased amount of explained variance in the spatial structure of EM fungi as tree mortality increased with subsequent increases in understory productivity and diversity and fine root biomass (Figs. 1A–1B and Fig. 2B). Although the amount of unexplained variation was high across all sites (>60%), compared to severely beetle-killed sites, undisturbed sites explained less of the variation in the spatial structuring of EM fungi. This may indicate that EM fungal community structure in undisturbed, even aged pine stands are more heterogeneous in both horizontal and vertical space, and are influenced by a complex array of interacting environmental conditions (Bahram, Peay & Tedersoo, 2015) in which no detectable spatial patterns in our study could be found. Alternatively, the lack of spatial structure in undisturbed versus severely beetle-killed sites could indicate fine-scale patterning below 0.5 m (Morris, 1999; Genney, Anderson & Alexander, 2006; Morris, Friese & Allen, 2007), or a greater role of dispersal limitation in undisturbed forested systems than previously anticipated (Lilleskov et al., 2004; Peay et al., 2012).

## Conclusions

Together, our results provide insight into how biotic factors determine the spatial structure of EM fungi and how large-scale disturbance increases the distance over which EM fungal communities exhibit spatial homogeneity. Understanding the underlying factors that contribute to the spatial structuring of EM fungal communities is of vital importance in order to make better predictions about the impact of bottom-up and top-down processes on (i) forest recovery, and (ii) the resilience of plant-microbe systems to environmental perturbations (Ettema & Wardle, 2002; Smith & Read, 2008; Simard, 2009).

##  Supplemental Information

10.7717/peerj.6895/supp-1 Figure S1 Representative site design for sampling of soil cores to determine changes in the spatial structuring of ectomycorrhizal fungal communities following recent mountain pine beetle activity (since 2009) across eleven sites in pine forests in west-central Alb A 40 m ×40 m plot was established within each of the eleven sites and ten 9 m ×9 m subplots within each plot. Within each of the subplots, eight soil cores were positioned at distances (0.5 m, 1 m, 1.5 m, 2 m, 3 m, 4 m, 5 m) randomly radiating from the center of each subplot.

10.7717/peerj.6895/supp-2 Table S1 Primer constructs used for Ion TorrentTM sequencing Ion Torrent TM adaptor, primer, and specific multiplex identifier (MID) barcode sequences.

10.7717/peerj.6895/supp-3 Table S2 A list of ectomycorrhizal (EM) fungal OTUs present in soil cores from undisturbed and severely beetled-killed (>60% Pinus contorta killed basal area) stands of west-central Alberta, Canada A CLAM analysis was performed to classify EM fungal OTUs into the following categories: EM fungi primarily found in undisturbed forest stands (Undisturbed), EM fungi primarily found in beetle-killed forest stands (Beetle-killed), and fungi common in both undisturbed and beetle-killed forest stands (Shared).

10.7717/peerj.6895/supp-4 Dataset S1 Data sets used in this study for determining the effects of biotic factors on the spatial structuring of ectomycorrhizal fungi Three data sets are shown, (i) raw OTU data table, (ii) final OTU data matrix, (iii) site-level properties matrix.

## Additional Information and Declarations

### DNA Deposition

The following information was supplied regarding the deposition of DNA sequences: Representative sequences of EM fungal OTUs are deposited in GenBank under accession numbers (KR584666–KR584685; KX497205–KX498025).

### Data Availability

The following information was supplied regarding data availability: The raw data has been supplied as Supplemental Information.
