# Supplementary figures and images for: Large-scale insect outbreak homogenizes the spatial structure of ectomycorrhizal fungal communities
Source: PeerJ. 2019 May 10;7:e6895. doi: 10.7717/peerj.6895 (PMC6512761; doi:10.7717/peerj.6895)

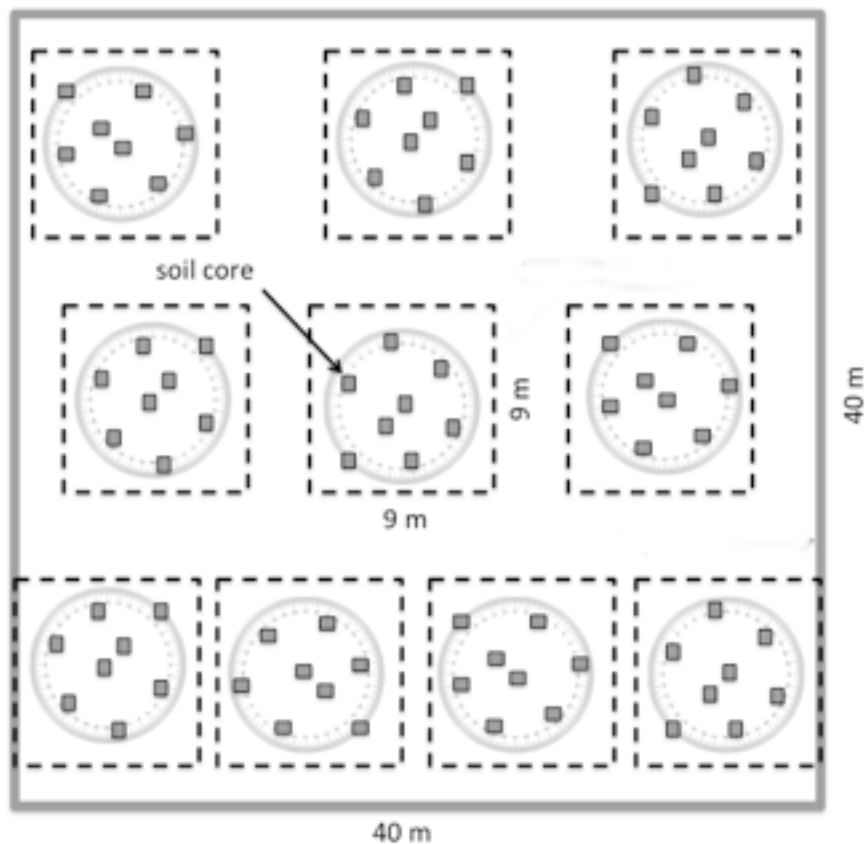

Supplement: Figure S1 — A 40 m ×40 m plot was established within each of the eleven sites and ten 9 m ×9 m subplots within each plot. Within each of the subplots, eight soil cores were positioned at distances (0.5 m, 1 m, 1.5 m, 2 m, 3 m, 4 m, 5 m) randomly radiating from the center of each subplot. [file peerj-07-6895-s001.pdf]
