# Supplementary material for: Large-scale insect outbreak homogenizes the spatial structure of ectomycorrhizal fungal communities
Source: PeerJ. 2019 May 10;7:e6895. doi: 10.7717/peerj.6895 (PMC6512761; doi:10.7717/peerj.6895)
Supplement: Table S1 — Ion Torrent TM adaptor, primer, and specific multiplex identifier (MID) barcode sequences. [file peerj-07-6895-s002.docx]

Table S1. Primer constructs used for Ion Torrent^TM^ sequencing

| **Ion Torrent** **^TM^** **adaptor** |  |
| --- | --- |
| Sequencing A adaptor | CAGTCGGGCGTCATCA |
| Sequencing trP1 adaptor | ATCACCGACTGCCCATAGAGAGG |
| **Gene Primer** |  |
| ITS1-F (forward) | CTTGGTCATTTAGAGGAAGTAA |
| ITS2 (reverse) | GCATCGATGAAGAACGCAGC |
| **Specific multiplex identifier barcode (MID) sequences** |  |
| CTAAGGTAAC | TTGAGCCTATTC |
| TAAGGAGAAC | CCGCATGGAAC |
| AAGAGGATTC | CTGGCAATCCTC |
| TACCAAGATC | CCGGAGAATCGC |
| CAGAAGGAAC | TCCACCTCCTC |
| CTGCAAGTTC | CAGCATTAATTC |
| TTCGTGATTC | TCTGGCAACGGC |
| TTCCGATAAC | TCCTAGAACAC |
| TGAGCGGAAC | TCCTTGATGTTC |
| CTGACCGAAC | TCTAGCTCTTC |
| TCCTCGAATC | TCACTCGGATC |
| TAGGTGGTTC | TTCCTGCTTCAC |
| TCTAACGGAC | CCTTAGAGTTC |
| TTGGAGTGTC | CTGAGTTCCGAC |
| TCTAGAGGTC | TCCTGGCACATC |
| TCTGGATGAC | CCGCAATCATC |
| TCTATTCGTC | TTCCTACCAGTC |
| AGGCAATTGC | TCAAGAAGTTC |
| TTAGTCGGAC | TTCAATTGGC |
| CAGATCCATC | CCTACTGGTC |
| TCGCAATTAC | TGAGGCTCCGAC |
| TTCGAGACGC | CGAAGGCCACAC |
| TGCCACGAAC | TCTGCCTGTC |
| AACCTCATTC | CGATCGGTTC |
| CCTGAGATAC | TCAGGAATAC |
| TTACAACCTC | CGGAAGAACCTC |
| AACCATCCGC | CGAAGCGATTC |
| ATCCGGAATC | CAGCCAATTCTC |
| TCGACCACTC | CCTGGTTGTC |
| CGAGGTTATC | TCGAAGGCAGGC |
| TCCAAGCTGC | CCTGCCATTCGC |
| TCTTACACAC | TTGGCATCTC |
| TTCTCATTGAAC | CTAGGACATTC |
| TCGCATCGTTC | CTTCCATAAC |
| TAAGCCATTGTC | CCAGCCTCAAC |
| AAGGAATCGTC | CTTGGTTATTC |
| CTTGAGAATGTC | TTGGCTGGAC |
| TGGAGGACGGAC | CCGAACACTTC |
| TAACAATCGGC | TCCTGAATCTC |
| CTGACATAATC | CTAACCACGGC |
| TTCCACTTCGC | CGGAAGGATGC |
| AGCACGAATC | CTAGGAACCGC |
| CTTGACACCGC | CTTGTCCAATC |
| TTGGAGGCCAGC | TCCGACAAGC |
| TGGAGCTTCCTC | CGGACAGATC |
| TCAGTCCGAAC | TTAAGCGGTC |
| TAAGGCAACCAC | CGGACAGATC |
| TTCTAAGAGAC | TTAAGCGGTC |
| TCCTAACATAAC |  |
| CGGACAATGGC |  |
