# Supplementary material for: Large-scale insect outbreak homogenizes the spatial structure of ectomycorrhizal fungal communities
Source: PeerJ. 2019 May 10;7:e6895. doi: 10.7717/peerj.6895 (PMC6512761; doi:10.7717/peerj.6895)
Supplement: Table S2 — A CLAM analysis was performed to classify EM fungal OTUs into the following categories: EM fungi primarily found in undisturbed forest stands (Undisturbed), EM fungi primarily found in beetle-killed forest stands (Beetle-killed), and fungi common in both undisturbed and beetle-killed forest stands (Shared). [file peerj-07-6895-s003.docx]

Table S2. A list of ectomycorrhizal (EM) fungal OTUs present in soil cores from undisturbed and severely beetled-killed (>60% Pinus contorta killed basal area) stands of west-central Alberta, Canada. A CLAM analysis was performed to classify EM fungal OTUs into the following categories: EM fungi primarily found in undisturbed forest stands (Undisturbed), EM fungi primarily found in beetle-killed forest stands (Beetle-killed), and fungi common in both undisturbed and beetle-killed forest stands (Shared).

| **Taxonomic**  **affiliation** | **Phylum** | **Order** | **Classification**  **category** |
| --- | --- | --- | --- |
| \| *Amphinema* \| \| --- \| \| *Russula* \| \| *Cortinarius* \| \| *Russula__1* \| \| *Piloderma* \| \| *Cortinarius__1* \| \| *Cortinarius__2* \| \| *Russulaceae* \| \| *Cortinarius__3* \| \| *Cortinarius__4* \| \| *Cortinarius__5* \| \| *Suillus* \| \| *Russula__2* \| \| *Russula__3* \| \| *Cortinarius__6* \| \| *Hymenogaster* \| \| *Cortinarius__7* \| \| *Cortinarius__8* \| \| *Pseudotomentella* \| \| *Tomentella* \| \| *Cortinarius__9* \| \| *Russula__4* \| \| *Cortinarius__10* \| \| *Russula__5* \| \| *Cortinarius__11* \| \| *Tomentella__1* \| \| *Hygrophorus* \| \| *Russulaceae__1* \| \| *Russulaceae__2* \| \| *Russula__6* \| \| *Russula__7* \| \| *Russula__8* \| \| *Russula__9* \| \| *Cortinarius__12* \| \| *Russula__10* \| \| *Amphinema__1* \| \| *Tylospora* \| \| *Inocybaceae* \| \| *Russula__11* \| \| *Russula__12* \| \| *Gymnomyces* \| \| *Rhizopogon* \| \| *Pseudotomentella__1* \| \| *Piloderma__1* \| \| *Cortinarius__13* \| \| *Cortinarius__14* \| \| *Cortinarius__15* \| \| *Russula__13* \| \| *Piloderma__2* \| \| *Cortinarius__16* \| \| *Cortinarius__17* \| \| *Cortinarius__18* \| \| *Russula__14* \| \| *Tomentella__2* \| \| *Wilcoxina* \| \| *Inocybaceae__1* \| \| *Russula__15* \| \| *Hygrophorus__1* \| \| *Wilcoxina__1* \| \| *Tylospora__1* \| \| *Russula__16* \| \| *Cortinarius__19* \| \| *Cortinarius__20* \| \| *Tomentella__3* \| \| *Amphinema__2* \| \| *Cortinarius__21* \| \| *Tylospora__2* \| \| *Piloderma__3* \| \| *Sebacinaceae* \| \| *Inocybaceae__2* \| \| *Cortinarius__22* \| \| *Piloderma__4* \| \| *Tomentella__4* \| \| *Tomentella__5* \| \| *Suillus__1* \| \| *Russula__17* \| \| *Cortinarius__23* \| \| *Piloderma__5* \| \| *Piloderma__6* \| \| *Russula__18* \| \| *Cortinarius__24* \| \| *Piloderma__7* \| \| *Suillus__2* \| \| *Piloderma__8* \| \| *Tuber* \| \| *Piloderma__9* \| \| *Tomentella__6* \| \| *Russula__19* \| \| *Russulaceae__3* \| \| *Russula__20* \| \| *Tomentella__7* \| \| *Tylospora__3* \| \| *Russula__21* \| \| *Russula__22* \| \| *Cortinarius__25* \| \| *Cortinarius__26* \| \| *Tomentella__8* \| \| *Lactarius* \| \| *Russula__23* \| \| *Elaphomyces* \| \| *Piloderma__10* \| \| *Piloderma__11* \| \| *Piloderma__12* \| \| *Russula__24* \| \| *Piloderma__13* \| \| *Piloderma__14* \| \| *Russula__25* \| \| *Russula__26* \| \| *Russulaceae__4* \| \| *Cortinarius__27* \| \| *Cortinarius__28* \| \| *Lactarius__1* \| \| *Tylospora__4* \| \| *Sphaerosporella* \| \| *Piloderma__15* \| \| *Wilcoxina__2* \| \| *Tomentella__9* \| \| *Tomentella__10* \| \| *Tomentella__11* \| \| *Hygrophorus__2* \| \| *Tylospora__5* \| | \| Basidiomycota \| \| --- \| \| Basidiomycota \| \| Basidiomycota \| \| Basidiomycota \| \| Basidiomycota \| \| Basidiomycota \| \| Basidiomycota \| \| Basidiomycota \| \| Basidiomycota \| \| Basidiomycota \| \| Basidiomycota \| \| Basidiomycota \| \| Basidiomycota \| \| Basidiomycota \| \| Basidiomycota \| \| Basidiomycota \| \| Basidiomycota \| \| Basidiomycota \| \| Basidiomycota \| \| Basidiomycota \| \| Basidiomycota \| \| Basidiomycota \| \| Basidiomycota \| \| Basidiomycota \| \| Basidiomycota \| \| Basidiomycota \| \| Basidiomycota \| \| Basidiomycota \| \| Basidiomycota \| \| Basidiomycota \| \| Basidiomycota \| \| Basidiomycota \| \| Basidiomycota \| \| Basidiomycota \| \| Basidiomycota \| \| Basidiomycota \| \| Basidiomycota \| \| Basidiomycota \| \| Basidiomycota \| \| Basidiomycota \| \| Basidiomycota \| \| Basidiomycota \| \| Basidiomycota \| \| Basidiomycota \| \| Basidiomycota \| \| Basidiomycota \| \| Basidiomycota \| \| Basidiomycota \| \| Basidiomycota \| \| Basidiomycota \| \| Basidiomycota \| \| Basidiomycota \| \| Basidiomycota \| \| Basidiomycota \| \| Ascomycota \| \| Basidiomycota \| \| Basidiomycota \| \| Basidiomycota \| \| Ascomycota \| \| Basidiomycota \| \| Basidiomycota \| \| Basidiomycota \| \| Basidiomycota \| \| Basidiomycota \| \| Basidiomycota \| \| Basidiomycota \| \| Basidiomycota \| \| Basidiomycota \| \| Basidiomycota \| \| Basidiomycota \| \| Basidiomycota \| \| Basidiomycota \| \| Basidiomycota \| \| Basidiomycota \| \| Basidiomycota \| \| Basidiomycota \| \| Basidiomycota \| \| Basidiomycota \| \| Basidiomycota \| \| Basidiomycota \| \| Basidiomycota \| \| Basidiomycota \| \| Basidiomycota \| \| Basidiomycota \| \| Ascomycota \| \| Basidiomycota \| \| Basidiomycota \| \| Basidiomycota \| \| Basidiomycota \| \| Basidiomycota \| \| Basidiomycota \| \| Basidiomycota \| \| Basidiomycota \| \| Basidiomycota \| \| Basidiomycota \| \| Basidiomycota \| \| Basidiomycota \| \| Basidiomycota \| \| Basidiomycota \| \| Ascomycota \| \| Basidiomycota \| \| Basidiomycota \| \| Basidiomycota \| \| Basidiomycota \| \| Basidiomycota \| \| Basidiomycota \| \| Basidiomycota \| \| Basidiomycota \| \| Basidiomycota \| \| Basidiomycota \| \| Basidiomycota \| \| Basidiomycota \| \| Basidiomycota \| \| Ascomycota \| \| Basidiomycota \| \| Ascomycota \| \| Basidiomycota \| \| Basidiomycota \| \| Basidiomycota \| \| Basidiomycota \| \| Basidiomycota \| | \| Atheliales \| \| --- \| \| Russulales \| \| Agaricales \| \| Russulales \| \| Atheliales \| \| Agaricales \| \| Agaricales \| \| Russulales \| \| Agaricales \| \| Agaricales \| \| Agaricales \| \| Boletales \| \| Russulales \| \| Russulales \| \| Agaricales \| \| Agaricales \| \| Agaricales \| \| Agaricales \| \| Thelephorales \| \| Thelephorales \| \| Agaricales \| \| Russulales \| \| Agaricales \| \| Russulales \| \| Agaricales \| \| Thelephorales \| \| Agaricales \| \| Russulales \| \| Russulales \| \| Russulales \| \| Russulales \| \| Russulales \| \| Russulales \| \| Agaricales \| \| Russulales \| \| Atheliales \| \| Atheliales \| \| Agaricales \| \| Russulales \| \| Russulales \| \| Russulales \| \| Boletales \| \| Thelephorales \| \| Atheliales \| \| Agaricales \| \| Agaricales \| \| Agaricales \| \| Russulales \| \| Atheliales \| \| Agaricales \| \| Agaricales \| \| Agaricales \| \| Russulales \| \| Thelephorales \| \| Pezizales \| \| Agaricales \| \| Russulales \| \| Agaricales \| \| Pezizales \| \| Atheliales \| \| Russulales \| \| Agaricales \| \| Agaricales \| \| Thelephorales \| \| Atheliales \| \| Agaricales \| \| Atheliales \| \| Atheliales \| \| Sebacinales \| \| Agaricales \| \| Agaricales \| \| Atheliales \| \| Thelephorales \| \| Thelephorales \| \| Boletales \| \| Russulales \| \| Agaricales \| \| Atheliales \| \| Atheliales \| \| Russulales \| \| Agaricales \| \| Atheliales \| \| Boletales \| \| Atheliales \| \| Pezizales \| \| Atheliales \| \| Thelephorales \| \| Russulales \| \| Russulales \| \| Russulales \| \| Thelephorales \| \| Atheliales \| \| Russulales \| \| Russulales \| \| Agaricales \| \| Agaricales \| \| Thelephorales \| \| Russulales \| \| Russulales \| \| Eurotiales \| \| Atheliales \| \| Atheliales \| \| Atheliales \| \| Russulales \| \| Atheliales \| \| Atheliales \| \| Russulales \| \| Russulales \| \| Russulales \| \| Agaricales \| \| Agaricales \| \| Russulales \| \| Atheliales \| \| Pezizales \| \| Atheliales \| \| Pezizales \| \| Thelephorales \| \| Thelephorales \| \| Thelephorales \| \| Agaricales \| \| Atheliales \| | \| Shared \| \| --- \| \| Shared \| \| Shared \| \| Shared \| \| Shared \| \| Undisturbed \| \| Shared \| \| Shared \| \| Undisturbed \| \| Shared \| \| Shared \| \| Undisturbed \| \| Shared \| \| Shared \| \| Shared \| \| Shared \| \| Shared \| \| Shared \| \| Shared \| \| Undisturbed \| \| Shared \| \| Undisturbed \| \| Shared \| \| Shared \| \| Shared \| \| Undisturbed \| \| Beetle-killed \| \| Shared \| \| Undisturbed \| \| Shared \| \| Shared \| \| Undisturbed \| \| Undisturbed \| \| Shared \| \| Shared \| \| Shared \| \| Shared \| \| Shared \| \| Shared \| \| Shared \| \| Undisturbed \| \| Undisturbed \| \| Shared \| \| Shared \| \| Shared \| \| Shared \| \| Shared \| \| Shared \| \| Shared \| \| Shared \| \| Undisturbed \| \| Shared \| \| Shared \| \| Shared \| \| Shared \| \| Shared \| \| Undisturbed \| \| Shared \| \| Shared \| \| Beetle-killed \| \| Shared \| \| Shared \| \| Shared \| \| Shared \| \| Undisturbed \| \| Undisturbed \| \| Shared \| \| Shared \| \| Shared \| \| Undisturbed \| \| Shared \| \| Shared \| \| Shared \| \| Beetle-killed \| \| Shared \| \| Shared \| \| Shared \| \| Beetle-killed \| \| Shared \| \| Shared \| \| Shared \| \| Shared \| \| Shared \| \| Shared \| \| Shared \| \| Shared \| \| Beetle-killed \| \| Shared \| \| Beetle-killed \| \| Undisturbed \| \| Undisturbed \| \| Beetle-killed \| \| Shared \| \| Shared \| \| Undisturbed \| \| Shared \| \| Shared \| \| Shared \| \| Undisturbed \| \| Shared \| \| Shared \| \| Beetle-killed \| \| Shared \| \| Beetle-killed \| \| Shared \| \| Shared \| \| Beetle-killed \| \| Shared \| \| Beetle-killed \| \| Shared \| \| Beetle-killed \| \| Shared \| \| Shared \| \| Shared \| \| Shared \| \| Shared \| \| Shared \| \| Shared \| \| Shared \| \| Beetle-killed \| \| Shared \| |
|  |  |  |  |
|  |  |  |  |
